# Vaccine subtype and dose interval determine immunogenicity of primary series COVID-19 vaccines in older people

**DOI:** 10.1016/j.xcrm.2022.100739

**Published:** 2022-08-25

**Authors:** Helen Parry, Rachel Bruton, Reni Ayodele, Penny Sylla, Graham McIlroy, Nicola Logan, Sam Scott, Sam Nicol, Kriti Verma, Christine Stephens, Brian Willett, Jianmin Zuo, Paul Moss

**Affiliations:** 1 Institute of Immunology and Immunotherapy, University of Birmingham, Birmingham B15 2TT, UK; 2 Institute of Cancer and Genomic Sciences, University of Birmingham, Birmingham B15 2TT, UK; 3 MRC-University of Glasgow Centre for Virus Research, University of Glasgow, Glasgow G61 1QH, UK

**Keywords:** COVID-19, vaccines, elderly, primary series, BNT162b2, ChAdOx1

## Abstract

Age is the strongest determinant of COVID-19 mortality, and over 2 billion people have received primary series vaccination with BNT162b2 (mRNA) or ChAdOx1 (adenoviral vector). However, the profile of sustained vaccine immunogenicity in older people is unknown. Here, we determine spike-specific humoral and cellular immunity to 8 months following BNT162b2 or ChAdOx1 in 245 people aged 80–98 years. Vaccines are strongly immunogenic, with antibodies retained in every donor, while titers fall to 23%–26% from peak. Peak immunity develops rapidly with standard interval BNT162b2, although antibody titers are enhanced 3.7-fold with extended interval. Neutralization of ancestral variants is superior following BNT162b2, while neutralization of Omicron is broadly negative. Conversely, cellular responses are stronger following ChAdOx1 and are retained to 33%–60% of peak with all vaccines. BNT162b2 and ChAdOx1 elicit strong, but differential, sustained immunogenicity in older people. These data provide a baseline to assess optimal booster regimen in this vulnerable age group.

## Highlights

• Dual COVID-19 vaccination with BNT162b2 or ChAdOx1 is immunogenic in older people
• Extending BNT162b2 dose interval increases antibody level but limits cellular response
• BNT162b2 gives higher antibody level, and ChAdOx1 stimulates strong T cell response
• Immune responses fall by 41%–76% at 8 months, less than reported in younger people

## Graphical abstract

**Figure:**
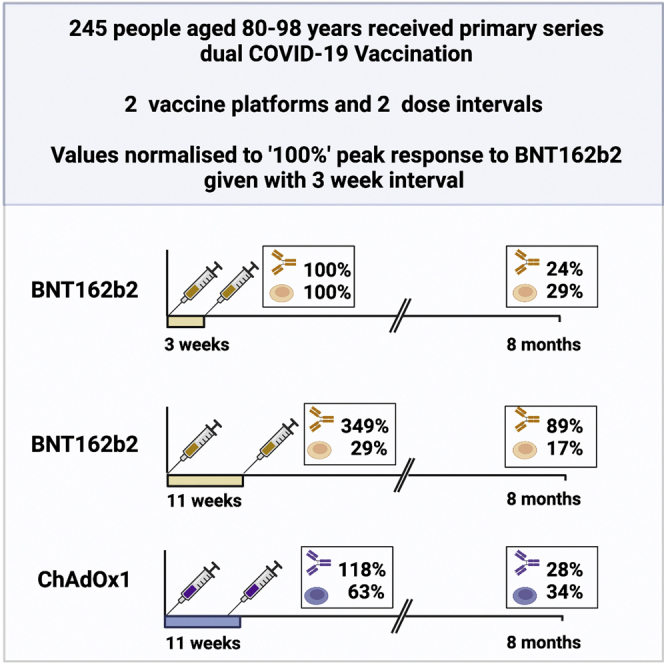

## Teaser

Dual “primary series” SARS-CoV-2 vaccination is the core of global vaccine protection. Parry et al. determine an 8-month profile of antibody and cellular immunity in people aged >80 years following mRNA or adenovirus vaccination. Both are immunogenic, but subtype and dose interval elicit differential immune platforms for booster vaccines.

## Introduction

The severe acute respiratory syndrome coronavirus 2 (SARS-CoV-2) pandemic is estimated to have led to the death of over 18 million people to date,^1^ and age is the strongest determinant of risk.^2^ Indeed, the median age of death within the first 6 months of the pandemic within the UK was 83 years.^3^ SARS-CoV-2 vaccines have transformed control of the COVID-19 pandemic and provide strong protection against severe disease and mortality.^4^, ^5^, ^6^ As such, there is considerable interest in the immunogenicity and clinical utility of vaccines in older population, and although older people were underrepresented within vaccine registration studies, a number of studies have demonstrated robust short term immunogenicity^7^ and are likely to underpin the strong clinical protection following vaccination observed in this group.^8^

Despite this, the longer-term immunogenicity of COVID-19 vaccines in this vulnerable population requires further investigation. In particular, there remains uncertainty regarding the potential importance of immune senescence on the magnitude and relative waning of vaccine responses in the very elderly.^9^ Initial reports indicate that spike-specific antibody titer can fall by over 18-fold from peak within 6 months,^10^ and such a rate of decline could be particularly important in those people with somewhat lower peak responses. Furthermore, clinical protection against vaccine breakthrough is likely to depend on the balance of humoral and cellular immunity, and while most studies have focused on antibody responses, it is now recognized that cellular immunity provides an essential component of immune protection.^11^ Vaccine subtypes employ differential mechanisms of delivery of the spike protein that are likely to influence the profile of spike-specific immune response, and previous studies early after the first^12^ and second dose^7^ have shown that mRNA vaccines elicit higher humoral immunity in older people, while adenoviral delivery can enhance cellular responses.^13^^,^^14^ A further influence on immunogenicity will relate to the time interval between the first and second doses of the dual vaccination. The BNT162b2 vaccine is generally delivered with a 3-week interval, but an extended 10- to 12-week interval has been used in some countries in order to maximize delivery of at least 1 vaccine across the population. This extended dose interval is also the standard approach for the ChAdOx1 primary series. Several reports have now shown that the extended interval regimen enhances mRNA-induced humoral immunity after the second dose, but this leaves a 10-week period of single-dose immunity during which low titer antibody responses are observed.^15^, ^16^, ^17^, ^18^ In contrast, the standard regimen induces a rapid immune response, although peak titers remain lower than observed following use of an extended interval regime.

Most COVID-19 vaccines comprise a two-dose primary series regimen, and although booster vaccines have been implemented in some settings, it remains critical to understand longer-term immunogenicity after the primary series, as this provides the platform on which boosters operate. Furthermore, booster vaccines have not been recommended in many countries, and compliance for acceptance of a third dose vaccine is far from complete. As such, primary series vaccination provides the platform for global immune protection. Here, we provide a detailed prospective assessment on spike-specific immune responses up to 8 months following 3 different COVID-19 vaccine regimens in older people and compare this profile with that seen at younger ages. Differential responses are seen in relation to vaccine subtype and dose interval, which provide insights that should help to guide future vaccine policy.

## Results

### Antibody responses are retained in all donors and titers increased 3.7-fold with extended interval mRNA vaccine

**Figure 1:**
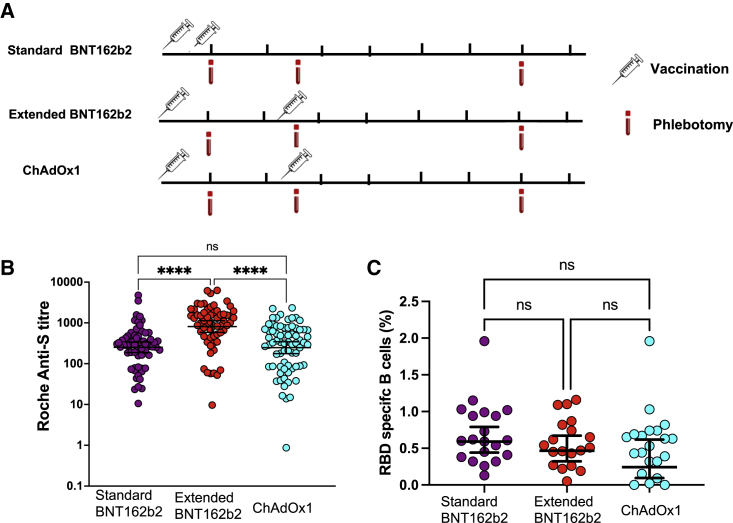
Spike-specific antibody titers at 8 months are enhanced following extended interval BNT162b2 vaccine regimen (A) Schematic representation of vaccine delivery within study groups. Three different cohorts of vaccinees were studied: 3-week, standard-interval BNT162b2 (sBNT162b2); 11-week, extended-interval BNT162b2 (eBNT162b2), or ChAdOx1 (11-week interval). Blood samples were taken at (1) 5 weeks post first vaccine (2 weeks after second dose for sBNT162b2 and 5 weeks after first dose for other two cohorts); (2) 13 weeks (10 weeks after second vaccine in sBNT162b2 cohort and 2 weeks after second dose for other two cohorts), and (3) 8 months. (B) Dot plot of spike-specific antibody responses in three cohorts at 8 months after the first vaccination (sBNT162b2 [n = 66], eBNT162b2 [n = 62], and ChAdOx1 [n = 72]). Statistical analysis with Kruskal-Wallis test followed by multiple comparisons. (sBNT162b2 versus eBNT162b2 p < 0.0001 and eBNT162b2 versus ChAdOx1 p < 0.0001). (C) Dot plot to show percentage of spike RBD-specific B cells as a proportion of the memory B cell population at 13–14 weeks post first dose of vaccine. Statistical analysis by Kruskal-Wallis test followed by multiple comparisons (sBNT162b2 0.7%, n = 20; eBNT162b2 0.6%, n = 19, and ChAdOx1 0.5%, n = 21).

Blood samples were taken from three groups of older donors living independently in the UK who had undergone dual COVID-19 vaccination with the first vaccine given 8 months previously. All donors were seronegative at each sample time point for previous SARS-CoV-2 infection, as determined by anti-nucleocapsid response, and none had received third booster vaccines. There were no cases of breakthrough infection identified in the 8 months follow up following the first vaccine dose. Two cohorts had received homologous BNT162b2 mRNA vaccination with either a standard 3-week or extended 11-week interval between the two doses. The third had received homologous ChAdOx1 vaccination with an 11-week interval between doses (Figure 1A).

Initial studies were undertaken 8 months following the first dose to determine spike-specific antibody titers within the three study groups using the Roche ELECCYS quantitative assay. Median values were 290 AU/mL for donors following standard-interval BNT162b2 (n = 66), 1,069 AU/mL for those after extended-interval BNT162b2 (n = 62), and 329 AU/mL following ChAdOx1 (n = 72) (Figure 1B). As such, antibody levels after extended-interval BNT162b2 were 3.7- and 3.2-fold higher than standard-interval BNT162b2 or ChAdOx1, respectively (p ≤ 0.0001).

The relative proportion of SARS-CoV-2 spike-specific B cells recognizing the spike receptor-binding domain (RBD) was then determined using a spike-tetramer flow cytometric assay on a subgroup of participants (19 on extended-interval BNT162b2, 20 on standard-interval BNT162b2, and 21 who received ChAdOx1). No difference was observed in age or gender distribution within these subgroups (p = 0.2). Median values of spike tetramer-specific B cells were 0.7%, 0.6%, and 0.5% across the vaccine regimens, values comparable to those seen after natural infection at this time point,^19^^,^^20^ with no difference seen between groups (Figure 1C).

These data indicate that antibody titers following the standard BNT162b2 and ChAdOx1 regimes are equivalent at 8 months after primary vaccination in older people. However, values are 3.2- to 3.7-fold higher when an extended-interval BNT162b2 protocol is employed.

### Spike-specific neutralizing antibody activity at 8 months is superior following BNT162b2 mRNA vaccination

**Figure 2:**
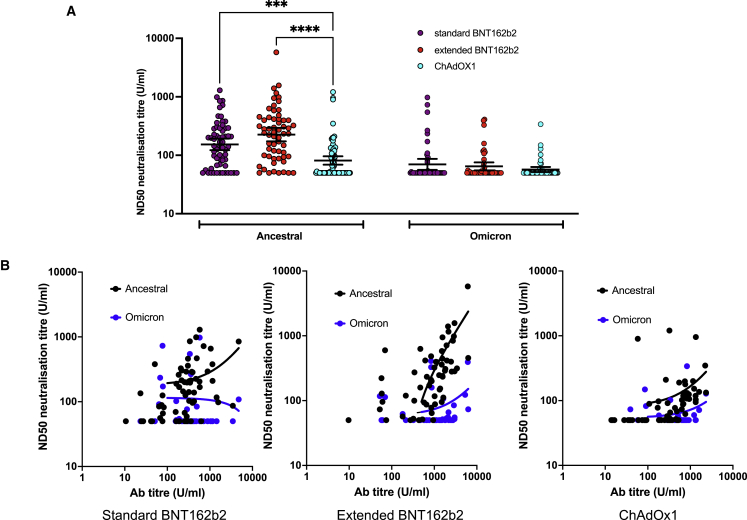
Viral neutralization activity within serum at 8 months post-vaccine is enhanced following BNT162b2 vaccination (A) Neutralization of HIV(SARS-CoV-2) pseudotypes bearing ancestral or Omicron spike glycoproteins by vaccine sera. ND50 calculated as reciprocal of dilution at which infectivity reduced to 50%. Dot plot shows ND50 from the three cohorts at 8 months post-vaccine. The lower limit of detection was >ND50, and values on the ND50 line are considered negative. Statistical difference analyzed by Kruskal-Wallis test followed by multiple comparisons. (sBNT162b2 versus ChAdOx1 p = 0.0006 and eBNT162b2 versus ChAdOx1 p < 0.0001). (B) Correlation between the spike-specific antibody titer and the pseudotype-derived ND50 against ancestral (red dot) or Omicron (black dot) between the three cohorts (ancestral: sBNT162b2 R = 0.48, p < 0.0001; eBNT162b2 R = 0.64, p ≤ 0.0001; ChAdOx1 R = 0.69, p < 0.0001; Omicron: sBNT162b2 R = −0.12, p = 0.41; eBNT162b2 R = 0.27, p = 0.07; ChAdOx1 R = 0.15, p = 0.31), with linear regression shown.

We next went on to assess the functional activity of spike-specific antibodies post-vaccination through measurement of neutralizing activity against ancestral and Omicron SARS-CoV-2 variants using HIV(SARS-CoV-2) pseudotypes. Reciprocal of serum dilution mediating 50% neutralization (ND50) titers against ancestral virus were 143 and 237 respectively following standard (n = 62) or extended-interval (n = 60) BNT162b2 vaccination. These compared to a value of 53 following ChAdOx1 (n = 73), indicating that neutralizing activity is relatively enhanced following mRNA vaccination (Figure 2A). Neutralization of Omicron was markedly impaired, with ND50 values <50 in the majority of donors, with no difference between vaccine cohorts.

We further determined the relationship between antibody titer and neutralizing activity to assess the relative functional activity of antibodies following each vaccination regimen. As expected, these values were correlated in all cases, although neutralization was somewhat suppressed compared with the antibody titer following ChAdOx1 vaccination (Figure 2B).

### Spike-specific cellular immune responses are enhanced following ChAdOx1 vaccination

An assessment of spike-specific cellular response following stimulation of peripheral blood mononuclear cells (PBMCs) with peptide pools from the spike protein of SARS-CoV-2 was next performed. The major focus was on interferon gamma (IFN-γ) release following peptide stimulation, and this was assessed by two platforms, ELISpot assay and plasma QuantiFERON concentration.

**Figure 3:**
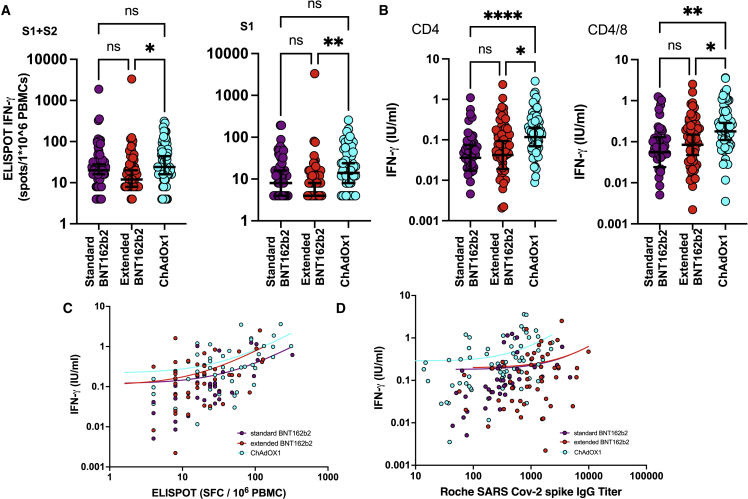
IFN-γ release following peptide stimulation is enhanced at 8 months following ChAdOx1 vaccination (A) Dot plot of spike-specific cellular responses measured by IFN-γ ELISpot within the three cohorts (spot-forming units [SFUs] per 10^6^ PBMCs). The left panel shows response following stimulation with total spike peptide pool (sBNT162b2 versus eBNT162b2 p = 0.12 and eBNT162b2 versus ChAdOx1 p = 0.01) and right panel shows response using spike S1 peptide pool (eBNT162b2 versus ChAdOx1 p = 0.001). Statistical analysis by Kruskal-Wallis test followed by multiple comparisons. (B) Dot plot of spike-specific cellular responses measured by QuantiFERON within the three cohorts. Data are shown as plasma concentration of IFN-γ (IU/mL) following peptide stimulation. The left panel shows the response following stimulation with HLA class II-binding peptides from the RBD region (sBNT162b2 versus ChAdOx1 p < 0.0001 and eBNT162b2 versus ChAdOx1 p = 0.01) and right panel shows response following stimulation of HLA class I- and class II-binding peptides from whole spike protein (sBNT162b2 versus ChAdOx1 p = 0.003 and eBNT162b2 versus ChAdOx1 p = 0.03). Statistical difference analyzed with Kruskal-Wallis test followed by multiple comparisons. (C) Correlation between ELISpot and QuantiFERON analysis following stimulation with using total spike peptide pool (sBNT162b2 R = 0.55, p = 0.0004; eBNT162b2 R = 0.53, p < 0.0001; ChAdOx1 R = 0.47, p = 0.0002) with linear regression line. (D) Correlation between spike-specific antibody titer and IFN-γ concentration from CD4/8 QuantiFERON assay (sBNT162b2 R = 0.68, p < 0.0001; eBNT162b2 R = 0.31, p = 0.02; ChAdOx1 R = 0.46, p < 0.0001) with linear regression line.

IFN-γ release was enhanced following ChAdOx1 vaccination in both assay systems. In particular, median ELISpot values following stimulation with peptides from the spike S1 domain were 14 spots/10^6^ in ChAdOx1 vaccinees (n = 72) compared with 8/10^6^ (n = 60) and 4/10^6^ (n = 57), respectively, following standard-or extended-interval BNT162b2 vaccination (p = 0.6 and 0.0013, respectively) (Figure 3A). Comparable values following stimulation with combined S1 and S2 peptide pools were 24, 20, and 12 across these three cohorts, respectively (ChAdOx1 versus extended BNT162b2, p = 0.012) (Figures 3A and S1). These data show that ELISpot responses are comparable between ChAdOx1 and standard-interval BNT162b2 vaccines but that values after extended-interval BNT162b2 vaccination are lower than ChAdOx1.

A more marked influence of vaccine platform was seen with the QuantiFERON assay where IFN-γ plasma concentration following whole-blood stimulation with HLA class II-binding peptides from RBD was 0.16 IU/mL following ChAdOx1 vaccination (n = 69), a value 3.2- and 2-fold higher than following standard- or extended-interval BNT162b2 vaccination, respectively (0.05 [n = 43] and 0.08 IU/mL [n = 55]) (Figure 3B). Comparable values following stimulation with combined CD4^+^ and CD8+-stimulatory peptides across spike were 0.20, 0.09, and 0.12 IU/mL, respectively, indicating a 2.2- and 1.7-fold increase within the ChAdOx1 group (Figure 3B).

ELISpot values and QuantiFERON IFN-γ concentration were correlated with a trend toward higher relative IFN-γ production following QuantiFERON stimulation in recipients of ChAdOx1 vaccine (Figure 3C). Assessment of QuantiFERON IFN-γ production in relation to spike-specific antibody titer also revealed a correlation within each cohort (Figure 3D).

These observations indicate that the adenovirus-based spike vaccine delivery elicits stronger IFN-γ response following peptide stimulation in older people at 8 months following dual vaccination.

### IL-2 release following spike peptide stimulation is increased following ChAdOx1 vaccination

SARS-CoV-2-spike-specific T cells are characterized by significant interleukin-2 (IL-2) production,^21^ with tumor necrosis factor (TNF) also produced early after natural infection.^22^ As such, we next went on to measure the concentration of IL-2 and TNF within QuantiFERON plasma samples following peptide stimulation. A LEGENDplex assay was used to measure IFN-γ, IL-2, and TNF concentrations within plasma samples, in the absence or presence of SARS-CoV-2 peptide stimulation, and these values were assessed as a ratio of increase following stimulation.

**Figure 4:**
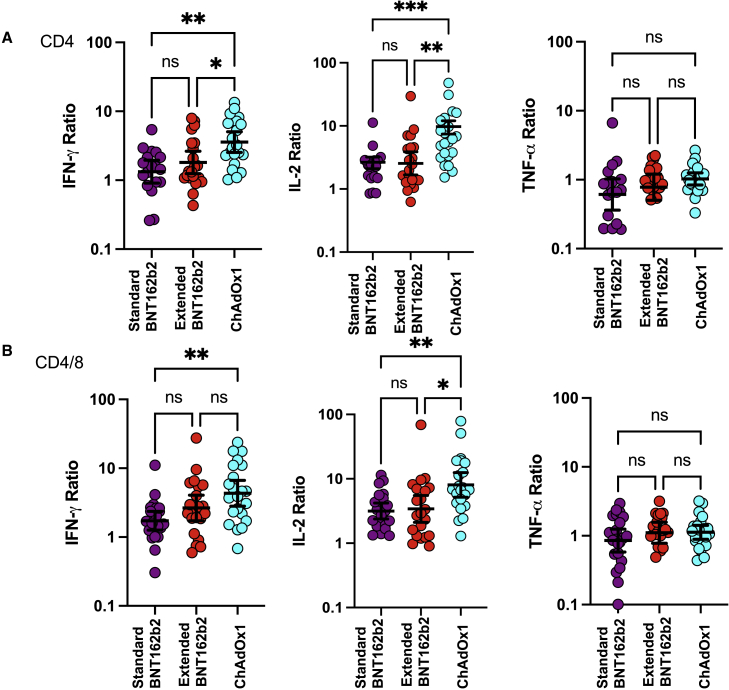
IL-2 production from spike-stimulated cells is relatively enhanced at 8 months following ChAdOx1 compared with BNT162b2 vaccination Legendplex technology was used to determine cytokine concentrations in plasma from QuantiFERON tubes following incubation in the absence or presence of spike peptides. The ratio of [cytokine] +peptide/−peptide is expressed on the y axis. (A) The top panels show the assay using CD4 T cell-specific peptides from the RBD region (IFN-γ: sBNT162b2 vs ChAdOx1 p=0.003 and eBNT162b2 vs ChAdOx1 p=0.03; IL-2: sBNT162b2 vs ChAdOx1 p=0.0007 and eBNT162b2 vs ChAdOx1 p=0.03). (B) Bottom panels show the assay using both CD4 T cell- and CD8 T cell-specific peptides from whole spike protein. The statistical difference was analyzed with Kruskal-Wallis test followed by multiple comparisons. (IFN-γ: sBNT162b2 versus ChAdOx1 p = 0.006; IL-2: sBNT162b2 versus ChAdOx1 p = 0.008 and eBNT162b2 versus ChAdOx1 p = 0.02).

IFN-γ ratios correlated strongly with the absolute plasma concentrations and, again, were higher in samples from ChAdOx1 vaccinees. In particular, values were 3.1 and 4.3 following stimulation with CD4 (RBD only) or CD4/8 peptides (whole spike), respectively, compared with 1.5 and 1.6 or 1.3 and 2.5 in recipients of BNT162b2 with either a standard- or extended-interval regime, respectively (CD4: ChAdOx1 versus s-BNT162b2, p = 0.003; ChAdOx1 versus e-BNT162b2, p = 0.025; CD4/8: ChAdOx1 versus s-BNT162b2, p = 0.0055; ChAdOx1 versus e-BNT162b2, p = 0.4) (Figures 4A and 4B).

IL-2 ratios were also markedly increased in recipients of the ChAdOx1 vaccine, with values of 6 and 6.8 following stimulation with CD4 or CD4/8 peptides, respectively, compared with 2.4 and 2.9 or 2 and 2.9 in recipients of BNT162b2 with either a standard- or extended-interval regime (CD4: ChAdOx1 versus s-BNT162b2, p = 0.0007; ChAdOx1 versus e-BNT162b2, p = 0.0026; CD4/8: ChAdOx1 versus s-BNT162b2, p = 0.0077; ChAdOx1 versus e-BNT162b2, p = 0.012) (Figures 4A and 4B). TNF values showed little increment following peptide stimulation and no variation in relation to the different vaccine regimens.

As such, the cellular response to spike peptide stimulation includes substantial IL-2 secretion at 8 months following each vaccine regimen, although this is enhanced following ChAdOX1.

### Antibody responses are comparable to those seen in adults aged between 59 and 80, while cellular responses are lower following BNT162b2

**Figure 5:**
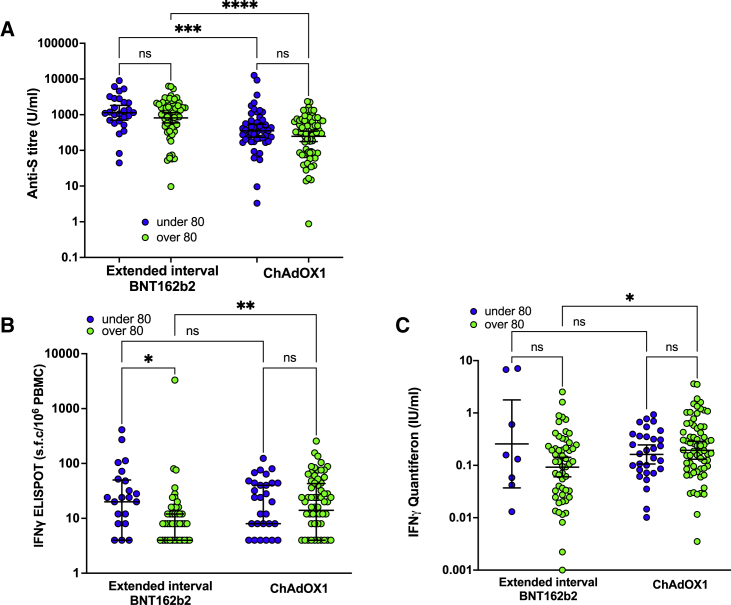
SARS-CoV-2-specific antibody responses in older donors are equivalent to younger people, but cellular responses are impaired following BNT162b2 vaccination Dot plots of spike-specific antibody and cellular responses at 8 months post-vaccination in donors <80 years (blue) and >80 years (green). (A) Spike-specific antibody titers (<80: n = 26 for BNT162, n = 48 for ChAdOx1; >80: n = 62 for BNT162b2, n = 72 for ChAdOx1) (for under 80, BNT162b2 versus ChAdOx1 p = 0.0006; for over 80, BNT162b2 versus ChAdOx1 p < 0.0001). (B) Spike-specific cellular response by IFN-γ ELISpot (SFUs/10^6^ PBMCs) (<80: n = 25 for BNT162, n = 40 for ChAdOx1; >80: n = 64 for BNT162b2, n = 72 for ChAdOx1) (for over 80, BNT162b2 versus ChAdOx1 p = 0.004; for BNT162b2, under 80 versus over 80 p = 0.013). (C) Spike-specific cellular response by plasma IFN-γ concentration following peptide stimulation in QuantiFERON assay (<80: n = 8 for BNT162, n = 30 for ChAdOx1; >80: n = 54 for BNT162b2, n = 68 for ChAdOx1) (for over 80, BNT162b2 versus ChAdOx1 p = 0.03). Median age of cohorts (years): 69 (interquartile rate [IQR] 59–75) and 83 (IQR 82–88) for BNT162b2; 72 (IQR 70–73) and 83 (IQR 81–86) for ChAdOx1. Statistical difference was analyzed with Kruskal-Wallis test followed by multiple comparisons.

The relative importance of immune senescence in relation to SARS-CoV-2-specific vaccine responses is critical for assessment of future vaccine policy. We next compared vaccine responses with two additional cohorts of 26 individuals aged between 70 and 73 years old for ChAdOx1 vaccine and 48 individuals aged between 59 and 75 years old for BNT162b2 vaccine (Table 1). Of note, donors aged <80 years who had received BNT162b2 with a standard 3-week interval were not available for study as this regimen had been discontinued in favor of an extended-interval regime for these younger age groups (Figure 5A).

SARS-CoV-2-specific antibody titers were broadly equivalent in both cohorts at 8 months following extended-interval BNT162b2 vaccination. Values were also comparable following ChAdOx1 vaccination, although, as observed within donors aged over 80 years, antibody titers were lower than those seen after mRNA vaccination within both age groups.

Spike-specific cellular immune responses were next compared across the age groups using IFN-γ ELISpot and Quantiferon assays. ELISpot responses against spike S1 peptides pool following BNT162b2 vaccination were higher in donors aged <80 years compared with those >80 years (20 versus 4 spots/10^6^, p = 0.01) (Figures 5B and 5C). A trend was also observed toward a lower Quantiferon response in the older donors, but as only 8 donors were available for analysis in the <80 years cohort, this was not conclusive. In contrast, cellular responses after ChAdOx1 were comparable within the two age groups.

These data indicate that there is no evidence for an impact of extreme immune senescence on antibody responses following SARS-CoV-2 vaccination within this cohort, although reduced levels of cellular response are observed following BNT162b2 vaccination.

### A differential profile of humoral and cellular immunity is observed in relation to vaccine subtype and dose interval

**Figure 6:**
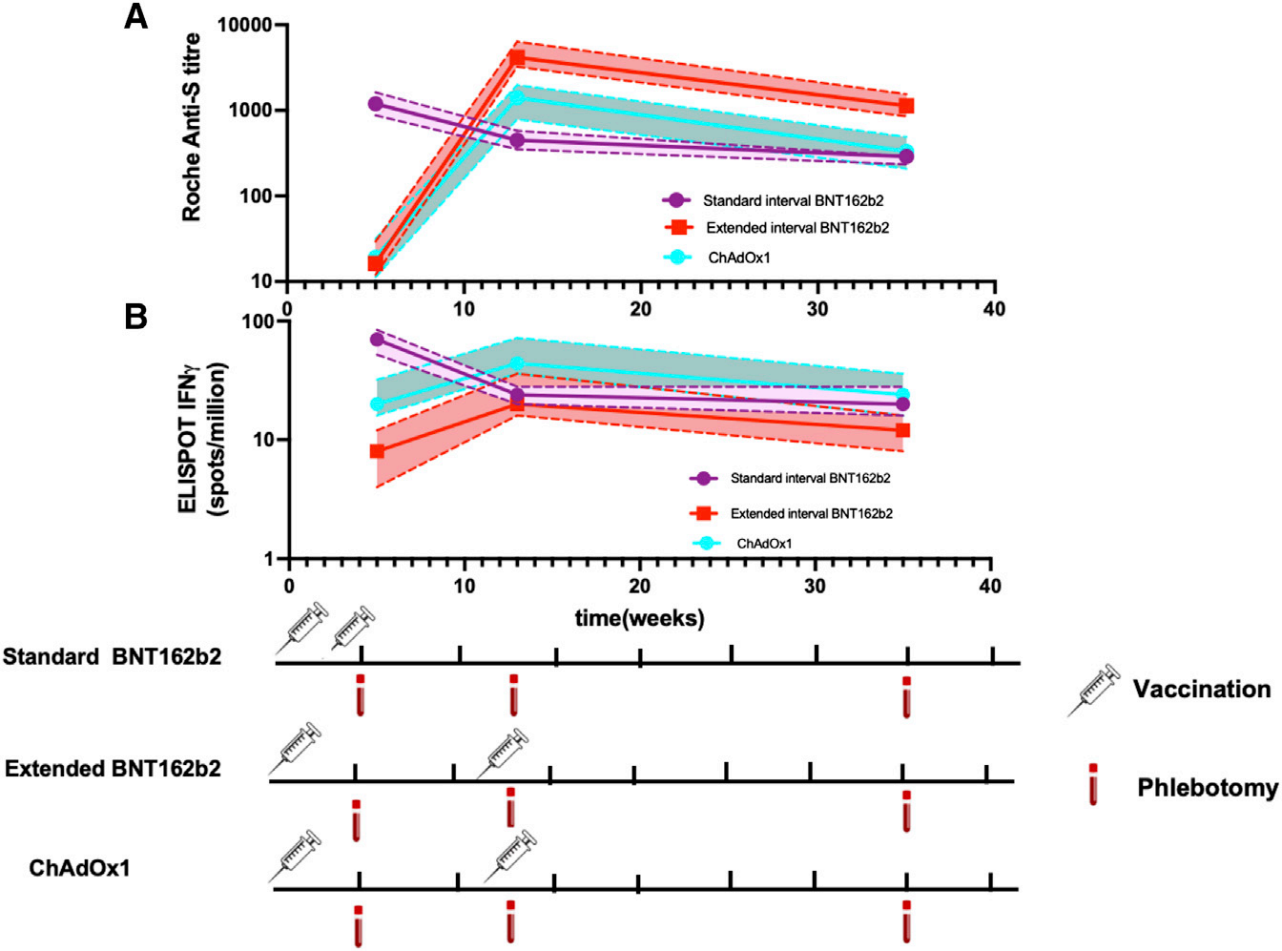
Profile of humoral and cellular immunity for 8 months following dual BNT162b2 or ChAdOx1 vaccination in donors aged >80 years (A) Spike-specific antibody titer at three time points serially collected among participants. Median titer with 95% confidence intervals (s-BNT162b2: 1,195, 450, and 290; e-BNT162b2: 16, 4,170, and 1,069; ChAdOx1: 19, 1,410, and 329). (B) Spike-specific cellular response at three time points. Median ELISPOT SFUs/10^6^ PBMCs with 95% confidence intervals (s-BNT162b2: 70, 24, and 20; e-BNT162b2: 8, 20, and 12; ChAdOx1: 20, 44, and 24). Number of samples, antibody: standard BNT162 n = 85, 74, and 62 at respective time points, extended BNT162b2 n = 69, 55, and 62, ChAdOx1 n = 84, 74, and 72; cellular: standard BNT162b2 n = 87, 77, and 60, extended BNT162b2 n = 72, 64, and 57, ChAdOx1 n = 85, 74, and 72.

In previous studies, we have reported on early antibody and cellular immunity following the first and second vaccine doses for participants in this study.^23^ As such, we combined these data with results at 8 months to determine the prospective profile of immune response following each vaccine platform (Figure 6).

**Table 1:** Patient demographics

	BNT162b2, 3 week, >80 years	BNT162b2, 11 week, >80 years	ChAdOx1, >80 years	BNT162b2, 11 week, <80 years	ChAdOx1, <80 years
Number	87	73	85	33	53
Median age	83	84	83	63	72
Age range	80–96	80–97	80–98	42–78	56–79
Age IQR	81–87	82–88	81–86	55–75	70–73
Male	37	33	34	14	20
Female	50	40	51	19	33

Standard 3-week-interval BNT162b2 vaccination produced an early rapid antibody rise, which then fell by 62% over 2 months before settling to a decline of 7.2% per month over the subsequent 5 months. As previously described, peak antibody responses are enhanced following extended interval BNT162b2 vaccination, and this increment was maintained over the following 5 months, with a decline of 15% per month. A comparable decline of 15% per month was also seen following ChAdOx1 vaccination.

A different profile was seen in relation to cellular responses where ELISpot analysis was available at each time point. Standard interval BNT162b2 vaccination induced a strong response at 70 spot-forming units (SFUs)/10^6^, which then demonstrated a biphasic fall of 33% and 3.4% per month over the subsequent 2 and 5 months, respectively. Extended-interval BNT162b2 vaccination elicited a weaker peak cellular response after the second vaccine, with a subsequent decline of 8% per month, and a similar kinetic profile was observed after ChAdOx1 vaccination, with a decline of 9% per month, although median values were higher both at the early and late time points after the second vaccine.

These data indicate that the immunogenicity of COVID-19 vaccines in older people is influenced substantially by both vaccine subtype and the time interval between doses.

## Discussion

Age is a strong determinant of clinical severity following SARS-CoV-2 infection,^24^ but this risk has been markedly reduced through the introduction of COVID-19 vaccines.^25^ Here, we undertook a detailed analysis of the vaccine immunogenicity in older people living in the community. This is arguably the most important demographic to study, as most COVID-19 deaths occur in elderly people and the great majority continue to live at home rather than in residential care. Furthermore, we studied BNT162b2 and ChAdOx1, two of the most widely utilized vaccines globally, which have been delivered to over 2 billion people to date. The results reveal a range of insights with importance for future global vaccine strategy.

Subjects were studied at 8 months after the first vaccine as this was the latest time point to assess responses following primary series vaccination, as a booster vaccine was recommended for older people at this point. Importantly, our analysis excluded people who had serological evidence of prior natural infection in order to assess true vaccine efficacy within a naive population. Interestingly, no subjects in any of the cohorts had evidence of breakthrough infection in the 8-month period following the first vaccine dose. This is likely to reflect vaccine efficacy and the “shielding” policy adopted by older people and recommended by the Department of Health in the UK during most of the study period. A striking feature was the impressive immunogenicity of both COVID-19 vaccines with SARS-CoV-2-specific antibody responses detected in every participant. Antibody titers were consistently higher following BNT162b2 vaccination compared with ChAdOx1,^26^^,^^27^ and mRNA delivery is known to generate intense humoral immune responses with prolonged local lymphadenopathy and germinal center formation.^28^ In addition, the BNT162b2 construct incorporates two proline residues that stabilize the spike protein in the pre-fusion conformation and may impact on the quality of immune response.^29^ Viral neutralization was also superior following mRNA vaccination. Spike-specific antibody levels and viral neutralization are emerging as correlates of immune protection,^30^^,^^31^ and these features are likely to underpin the impressive clinical efficacy of BNT162b2 in prevention of SARS-CoV-2 infection.

A further feature was that use of an extended time interval of 10–12 weeks between the two doses of BNT162b2 led to a sustained increase in the SARS-CoV-2-specific antibody titer, which was 3.7-fold higher at 8 months following vaccination. A comparable increase of 3.5-fold was observed at 2 weeks following the second dose,^18^ and as such, this enhancement is retained over the subsequent 5 months. Similar findings have been reported in younger adults at 4–8 weeks following the second vaccine,^16^^,^^17^ although the relative increment seems marked in older people. The immunological basis for this association is uncertain, but the sustained difference suggests that enhancement of spike-specific plasma cell generation is likely. Extended interval delivery might represent a viable option for delivery of the BNT162b2 vaccine,^32^ and an 8-week interval has recently been considered as potentially optimal for donors up to age 39 years in the United States.^33^ However, clinical efficacy has been excellent with the standard 3-week interval between doses,^34^ and T cell responses continued to be somewhat lower following an extended-interval regime, as seen at earlier time points.^16^^,^^18^ Furthermore, the standard interval also provides earlier peak antibody protection. Further research is now needed to assess how the extended-interval regime impacts on long-term humoral and cellular responses, and this work should extend beyond peripheral blood to study immunity at sites of viral replication such as mucosal surfaces within the respiratory tract.

A different pattern of immunogenicity was apparent in relation to spike-specific cellular responses, which showed relative enhancement following delivery of ChAdOx1 by several methods. Stronger cellular responses were seen previously at early time points following this vaccine,^12^ and here we show this is maintained up to 8 months. Median ELISpot responses were twice as high compared with the extended interval BNT162b2, although they were not significantly increased above standard BNT162b2 delivery. A similar finding was revealed in a review of vaccine immunogenicity,^13^ and the Ad26.COV2.S adenovirus-based vaccine also elicits particularly strong cellular responses.^14^ The Quantiferon SARS-CoV-2 assay^35^ was used for the first time in this cohort at the 8-month time point and also showed markedly increased IFN-γ release after peptide stimulation within ChAdOx1 vaccinees compared with both mRNA regimens.

QuantiFERON stimulation allows assessment of additional cytokine concentrations following peptide stimulation, and robust IL-2 responses were observed with each of the vaccine regimens, which augurs well for the generation and maintenance of spike-specific T cell memory. However, IL-2 production was >2-fold higher following ChAdOx1, indicating that production of both IFN-γ and IL-2 is enhanced following adenoviral-based vaccine delivery. The basis for this is uncertain, but the use of a viral vector may potentially enhance antigen presentation of spike peptides for cellular responses through infection of a fibroblast niche.^36^ In contrast to antibody neutralization, vaccine-induced cellular immunity is relatively preserved against SARS-CoV-2 variants,^37^ and recent data show excellent protection from severe Omicron infection in older donors who were primed with ChAdOx1 prior to an mRNA booster.^38^ Enhanced cellular responses following ChAdOx1 might be expected to translate into superior maintenance or affinity maturation of humoral responses,^39^ and a somewhat lower rate of spike-specific antibody decline following ChAdOx1 vaccination has been reported.^26^ However, this pattern was not observed within this cohort, and antibody neutralization activity was somewhat lower following this regimen.

The availability of samples at earlier time points from each participant allowed comparison of the kinetics of antibody and cellular responses between the three vaccine regimens. In recipients of standard-interval BNT162b2, we had access to a blood sample between the peak response and the 8-month assessment. This revealed an early rapid decline in antibody and cellular response over the first 2 months after the second vaccine, as seen in younger donors,^10^ before immune waning stabilized over the subsequent 5 months, with reductions of 7.2% and 3.4% per month in antibody titer and cellular response, respectively. The median antibody titer at 8 months was 24% of that seen at peak, a less marked reduction than reported in younger people,^10^^,^^40^^,^^41^ while cellular responses fell to 33% of peak value.

A notable feature was that the kinetics of antibody and cellular responses were almost identical following BNT162b2 or ChAdOx1 when an extended dose interval of 11 weeks was employed. The rates of antibody and cellular decline after peak response, at 15% and 8%–9% each month, respectively, were very similar, although differences were seen in absolute values. The lack of sampling in the 5-month period between peak response and 8-month assessment did not permit intermediate time point analysis of immune waning. However, median antibody titers at 8 months remained at 26% and 23% of those seen at peak for the extended BNT162b2 and ChAdOx1 regimes, respectively, similar to the standard BNT162b2 regime. Importantly, this decline over 8 months is substantially less marked than the 17- to 29-fold decline reported for donors aged 30–42 years after mRNA vaccination.^14^ Cellular responses were retained at 60% and 55% of peak value, respectively, and in keeping with increased stability of long-term T cell memory.

Participants had a median age of 85 years, and while vaccine immunogenicity was impressive, we were interested in comparing responses with those seen in adults aged <80 years. Antibody responses were entirely comparable with people of a median age of 67 years, showing no impact of extreme aging on the magnitude of the humoral response. However, we did not compare these values with younger adults, where several reports have observed a difference in antibody responses compared with older people.^10^^,^^26^^,^^40^

A somewhat different profile was seen in relation to cellular immunity where reduced IFN-γ responses were seen in older people who had received the BNT162b2 vaccine.^42^ In contrast, no age-related decline was observed following ChAdOx1, and this is noteworthy in relation to previous reports that antibody responses following adenoviral-based vaccines are also less influenced by factors such as age and underlying health conditions compared with mRNA regimens.^26^ It is important to note that the age distributions of these control cohorts varied (59–75 for BNT162b2 and 70–73 years for ChAdOx1) and as such may present potential bias in the interpretation of the vaccine responses observed.

In conclusion, we show that BNT162b2 and ChAdOx1 vaccines are strongly immunogenic in older people and show substantial retention of antibody and cellular responses over at least 8 months. However, differential features of humoral and cellular immune responses are observed, and it will be important to assess how these will translate into long-term protection or modulate immunogenicity following booster vaccination.^43^

### Limitations of the study

Limitations include the fact that this was not a clinical trial but a real-world analysis of vaccine responses. Indeed, these donors were some of the first people in the world to receive vaccination as part of a national vaccine campaign. Demographic information on participants is limited but all are independently living. In addition, while antibody and ELISpot were recorded at three time points, the QuantiFERON assay was assessed only at the 8-month time point.

## STAR★Methods

### Key resources table

**Table:** 

REAGENT or RESOURCE	SOURCE	IDENTIFIER
**Antibodies**		

FITC conjugated anti-IgD	Biolegend	Cat # 348206; RRID:AB_10612567
PerCP5.5 conjugated anti-CD20	Biolegend	Cat # 302326; RRID:AB_893283
Pe-Cy7 conjugated anti-CD38	Biolegend	Cat # 303516;RRID:AB_2072782
AF700 conjugated anti-IgM	Biolegend	Cat # 314538;RRID:AB_2566615
APC-Cy7 conjugated anti-CD27	Biolegend	Cat # 356424;RRID:AB_2566773
BC510 conjugated anti-CD19	Biolegend	Cat # 302242;RRID:AB_2561668
Live dye	Thermo Fisher	Cat # L34955
PE Streptavidin	Biolegend	Cat # 405245
APC Streptavidin	Biolegend	Cat # 405207

**Recombinant DNA**		

SARS-CoV-2 spike gene expression vectors	Willett et al., 2022	N/A
lentiviral vectors p8.91	Davis et al., 2021	N/A
pCSFLW	Davis et al., 2021	N/A

**Chemicals, peptides, and recombinant proteins**		

Biotinylated spike RBD protein	Biolegend	Cat # 793906
Dulbecco’s Phosphate Buffered Saline	Sigma	Cat # D8537
Fetal Bovine Serum	Sigma	Cat # F9665
GlutaMax Supplement	Sigma	Cat # G7513
RPMI	Sigma	Cat # R8758
DMEM	Sigma	Cat # D5796
Penicillin-Streptomycin	Sigma	Cat # P4333
Spike peptide pool	JPT Peptide Technologies	Cat # PM-WCPV-S-2
Critical commercial assays		
Immunotec T-SPOT® SARS-CoV-2 assay	Oxford Immunotec Ltd	Cat # COV.435/300
QuantiFERON SARS-CoV-2 interferon-γ release assay	Qiagen	Cat # 626715
LEGENDplex™ COVID-19 Cytokine Storm Panel 1	Biolegend	Cat # 741090

**Experimental models: Cell lines**		

HEK293	ATCC	Cat # CRL-1573
HEK293T	ATCC	Cat # CRL-3216
293-ACE2	Willett et al., 2022	N/A

**Software and algorithms**		
GraphPad Prism	GraphPad	V9

### Resource availability

#### Lead contact

Further information and requests for resources and reagents should be directed to the lead contact, Professor Paul Moss (P.moss@bham.ac.uk).

#### Materials availability

All requests for resources and reagents should be directed to the lead contact author. This includes viruses. All reagents will be made available on request after completion of a Materials Transfer Agreement.

### Experimental model and subject detail

#### Patients and samples

Participants aged 80 years and older, and who were living independently, were invited to participate in the study and recruited between 29th December 2020 and 4th March 2021. Co-morbidities were permitted. Local primary care networks identified vaccinees aged 80 years and older who had received either the BNT162b2 or ChAdOx1 vaccines and who were not living in a residential or care home or requiring assisted living. Participants were either invited to participate on attending the local vaccination centres or sent invitation letters to take part. Following initial contact with the study team, participants were then given the participant information sheet and consented verbally over the phone. This was substantiated with written consent obtained at the first phlebotomy time point. Ethical approval was obtained from North West Preston Research Ethics Committee with favourable outcome (REC 20\NW\0240) and work was performed under the CIA UPH and conducted according to the Declaration of Helsinki and good clinical practice.

Home phlebotomy was organised for all participants and sample collection determined by participant availability at each time point. Donors were tested after each of the time points for evidence of SARS-CoV-2 infection, as determined by anti-nucleocapsid response and excluded from analysis if positive. None had received 3rd booster vaccines.

After excluding donors with natural infection, 87 donors received BNT162b2 on a 3 week dosing interval (median age 83 years, range 80–96; IQR 81–87; 37 male ((43%)); 73 donors received BNT162b2 on an extended interval (median age 84, range 80–97; IQR 82–88; 33 male (45%)) and 85 received ChAdOx1 (median age 83 years, range 80–98, IQR 81–86; 34 were male (40%)). Controls aged <80 were also recruited following identification from primary care records and a letter invitation (33 donors received the BNT162b2 on an extended interval (median age 63, range 42–78; IQR 55–75. 14 male (42%)) and 53 received the ChAdOx1 (median age 72 range 56–79 IQR 70–73. 20 male donors (38%)) (Demographics in Table 1).

#### Cells and viruses

For pseudoneutralisation assays HEK293, HEK293T and 293-ACE2 cells were maintained in Dulbecco’s modified Eagle’s medium (DMEM) supplemented with 10% fetal bovine serum, 200 mM L-glutamine, 100 μg/mL streptomycin and 100 IU/mL penicillin. 293-ACE2 target cells were maintained in complete DMEM supplemented with 2 μg/mL puromycin. HEK293T cells were transfected with the appropriate SARS-CoV-2 spike gene expression vector in conjunction with lentiviral vectors p8.91^44^ and pCSFLW (Davis C et al., 2021) using polyethylenimine (PEI, Polysciences, Warrington, USA). HIV (SARS-CoV-2) pseudotype-containing supernatants were harvested 48 h post-transfection, aliquoted and frozen at −80°C prior to use. The delta construct bore the following mutations relative to the ancestral Hu-1 sequence (GenBank: MN908947): T19R, G142D, E156del, F157del, R158G, L452R, T478K, D614G, P681R, D950N.

### Method details

#### Roche Elecsys® electrochemiluminescence immunoassay (ECLIA)

Total antibodies specific to SARS-CoV-2 were detected using electrochemiluminescence assays on the automated Roche cobas e801 analysers based at Public Health England (PHE) Porton. Calibration and quality control were performed as recommended by the manufacturer. Anti-nucleocapsid protein (NP) antibodies were detected using the qualitative Roche Elecsys® AntiSARS-CoV-2 ECLIA (COV2, Product code: 09203079190), whilst anti-spike (S) antibodies were detected using the quantitative Roche Elecsys® Anti-SARS-CoV-2 S ECLIA (COV2 S, Product code 09289275190. Anti-nucleocapsid results are expressed as cut-off index (COI) value, with a COI value of ≥1.0 considered positive for anti-nucleocapsid antibodies. Anti-spike results are expressed as units per mL (AU/mL), with samples with a result of ≥0.8 AU/mL considered positive for anti-spike antibodies within the fully quantitative range of the assay: 0.4–2,500 AU/mL. Samples >2,500 AU/mL were diluted further (1:100) to within the quantitative range.

#### Cellular assays

Peripheral blood mononuclear cells (PBMCs) were isolated from a whole blood sample using ‘T-Cell Xtend’ (Oxford Immunotec) and Ficoll. After quantification and dilution of recovered cells, 250,000 PBMC were plated into each well of a ‘T-SPOT Discovery SARS-CoV-2’ kit (Oxford Immunotec). This is designed to measure responses to overlapping peptides pools covering protein sequences of SARS-CoV-2 spike antigen. Negative control and PHA-stimulated cells as a positive control were included. Peptide sequences that showed high homology to endemic coronaviruses were removed from the sequences, but sequences that may have homology to SARS-CoV-1 were retained. Cells were incubated and interferon-γ secreting T cells were counted. The data was shown as spot forming units (SFU) per million PBMC.

#### Quantiferon assay

The T cell responses was also measured by quantiferon assay was carried out using the QuantiFERON SARS-CoV-2 assay (Qiagen). 1mL of whole blood was added to the test tubes, including QuantiFERON Nil, QFN SARS CoV-2 Ag1 and QFN SARS CoV-2 Ag2 tube. The first tube serves as negative control. The QFN SARS CoV-2 Ag1 and Ag2 tubes contain mixed epitopes either from Spike RBD region that are restricted through MHC-II or from whole spike that can stimulate both CD4 + and CD8 + T cells responses. After 18h incubation, plasma from all three test tubes are tested for IFN-γ using an enzyme-linked immunosorbent assay (ELISA)-based platform. The data was shown as concentration of IFN-γ in IU/mL after the deduction from the QFN-SARS-CoV-2 Nil tube.

#### Legendplex assay

The plasma sample from the Quantiferin Assay were assessed using a LEGENDplex™ COVID-19 Cytokine Storm Panel 1 (14-plex) (BioLegend) according to the manufacturer’s instructions. Briefly, the panel of capture beads are cocultured with the plasma samples before the biotinylated detection antibodies are added. At last the Streptavidin-phycoerythrin (SA-PE) is used to bind to the biotinylated detection antibodies. The samples were analysed flow cytometer and the concentration of the cytokine is determined according to the standard curve. Data were analysed with the LEGENDplex v.8.0 software. The data was shown as ratio of concentration of IFN-γ compare with that from the QFN-SARS-CoV-2 Nil tube.

#### B cell tetramer staining

To detect SARS-CoV-2 specific B cells, biotinylated spike RBD protein antigen (Biolegend) was multimerized with PE and APC labeled streptavidin. PBMC samples were stained with these two Spike tetramers before the surface antibodies were added. The data were acquired using Beckman Coulter Gallios Flow Cytometer and analysed with kaluza software.

The frequency of spike-specific memory B cells was expressed as a percentage of total memory B cells population.

#### Pseudotype-based neutralization assays

HEK293T cells were transfected with the SARS-CoV-2 S gene expression vector in conjunction with packaging lentiviral vectors to generate Human immunodeficiency virus (HIV) (SARS-CoV-2) pseudotype. Neutralizing activity in each sample was measured using serial dilution in triplicate from 1:50 to 1:36,450. After the incubation of HIV (SARS-CoV-2) pseudotypes with serum sample, they were plated onto 239-ACE2 target cells. After 48–72 h, luciferase activity was quantified on a PerkinElmer EnSight multimode plate reader. Antibody titer was then calculated by interpolating the point at which infectivity had been reduced to 90% of the value for the no serum control samples.

### Quantification and statistical analysis

Data were tested for normality using Kolmogorov-Smirnov analysis. For comparative analysis of antibody titres and cellular responses between the cohorts, Kruskal-wallis with post hoc Dunn analysis was performed. The correlation was carried out using nonparametric Spearman’s rank-order correlation test. Antibody titres and T cell responses are presented as the median and IQR. 1. Statistical significance shown as: p < 0.05(∗), p < 0.01(∗∗), p < 0.001(∗∗∗) and p < 0.0001(∗∗∗∗). All analysis was performed using Graphpad prism v9.1.0 for Mac (San Diego, California USA).

## Data Availability

• Data. All data (including raw data used to generate neutralizing and binding curves) reported in this paper will be shared by the lead contact upon request. • Code. This paper does not report original code. • Additional information. Any additional information required to reanalyze the data reported in this paper is available from the lead contact upon request.

## Supplemental information

Document S1. Figure S1

Document S2. Article plus supplemental information
