# Supplementary material for: Vaccine subtype and dose interval determine immunogenicity of primary series COVID-19 vaccines in older people
Source: Cell Rep Med. 2022 Aug 25;3(9):100739. doi: 10.1016/j.xcrm.2022.100739 (PMC9404227; doi:10.1016/j.xcrm.2022.100739)
Supplement: Document S1. Figure S1 [file mmc1.pdf]

Cell Reports Medicine, Volume *3*

Supplemental information

Vaccine subtype and dose interval determine immunogenicity of primary series COVID-19 vaccines in older people

Helen Parry, Rachel Bruton, Reni Ayodele, Penny Sylla, Graham McIlroy, Nicola Logan, Sam Scott, Sam Nicol, Kriti Verma, Christine Stephens, Brian Willett, Jianmin Zuo, and Paul Moss

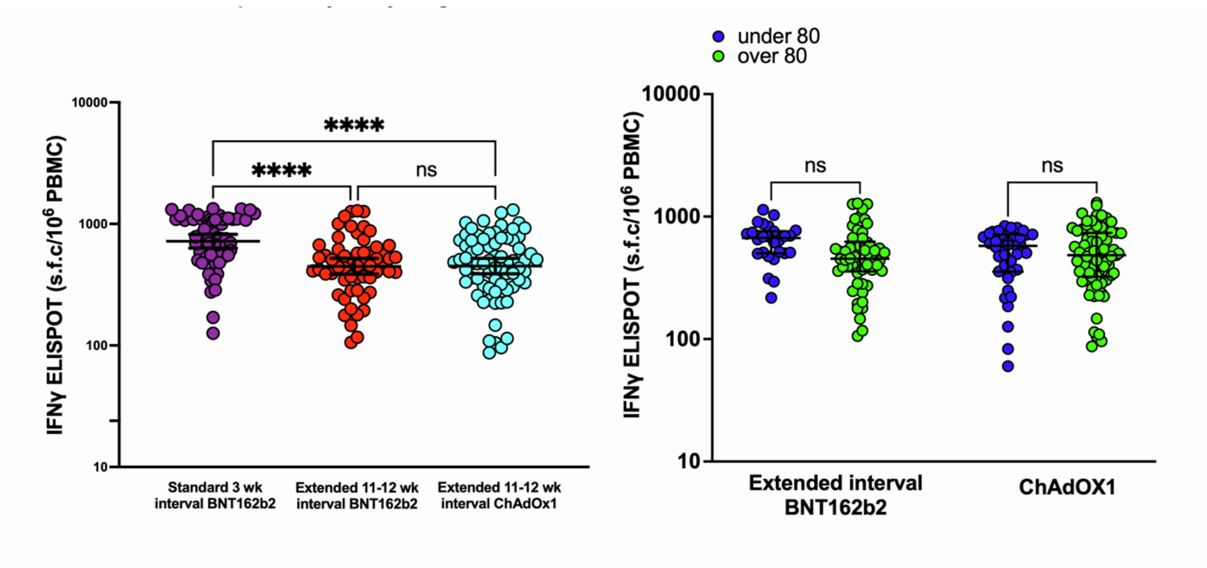

Supplementary Figure 1

PHA-stimulated positive control values for ELISpot analysis in vaccine cohorts.

(A) PHA responses in three vaccine cohorts at 8 months following first vaccination

(B) Comparative PHA responses in cohorts >80 and <80 years of age at 8 months following first vaccination.

Related to Figure 3.
